# Clinicians’ Attitudes Toward Telepsychology in Addiction and Mental Health Services, and Prediction of Postpandemic Telepsychology Uptake: Cross-sectional Study

**DOI:** 10.2196/35535

**Published:** 2022-05-13

**Authors:** Kristen Zentner, Graham Gaine, Paige Ethridge, Shireen Surood, Adam Abba-Aji

**Affiliations:** 1 Clinical Supports and Services Addiction & Mental Health Alberta Health Services Edmonton, AB Canada; 2 Department of Educational Psychology University of Alberta Edmonton, AB Canada; 3 Department of Psychiatry University of Alberta Edmonton, AB Canada; 4 Department of Psychology Glenrose Rehabilitation Hospital Alberta Health Services Edmonton, AB Canada; 5 Young Adult Assessment, Evaluation & Reintegration Addiction and Mental Health Alberta Health Services Edmonton, AB Canada

**Keywords:** mental health, telepsychology, clinician attitude, unified theory of acceptance and use of technology, therapeutic alliance

## Abstract

**Background:**

The COVID-19 pandemic has resulted in unprecedented uptake of telepsychology services; however, clinicians have mixed attitudes toward virtual technologies.

**Objective:**

This study (1) explored clinicians’ experiences of and intentions to use video, telephone, and in-person services, and (2) tested the utility of the unified theory of acceptance and use of technology (UTAUT) to predict clinicians’ intentions to offer telepsychology after the COVID-19 pandemic.

**Methods:**

Clinician satisfaction and therapeutic alliance were compared across in-person, video, and telephone services, while technology attitudes and intention to use after the pandemic were compared across video and telephone services among 118 addiction and mental health clinicians during the COVID-19 pandemic.

**Results:**

Clinicians reported more positive experiences with in-person services than both virtual technologies; further, clinicians reported greater positive experiences, attitudes, and intentions to use video services than telephone services across measures. Based on the UTAUT, performance expectancy positively predicted concurrent intentions to use video services (*β*=0.46; *P*<.001) and telephone services (*β*=0.35; *P*<.001) after the pandemic. Social influence (*β*=0.24; *P*=.004) and facilitating conditions (*β*=0.19; *P*=.03) additionally predicted the intention to use telephone services.

**Conclusions:**

Clinicians rated in-person services more positively than virtual technologies, with video services perceived more positively than telephone services. Performance expectancy was the primary facilitator of the uptake of both virtual modalities.

## Introduction

The COVID-19 pandemic has triggered an unprecedented shift toward virtual health care delivery [1]. Telepsychology is the provision of addiction and mental health (AMH) care from a physical distance and includes psychiatric evaluations, therapy, psychoeducation, and medication management [2]. While evidence supports the effectiveness of telepsychology [3-7], clinician hesitancy impedes uptake [5,8,9]. Because telepsychology is expected to play a continued role in service delivery after the pandemic [1,10,11], it is critical to understand clinicians’ perceptions of and attitudes toward telepsychology.

A clinician’s experience with telepsychology may also contribute to uptake. Perceived weakened therapeutic alliance may be a contributor to poor uptake of telepsychology [4,5], with therapists perceiving poorer therapeutic alliance in virtual than in-person settings [12]. In contrast, client ratings of therapeutic alliance are comparable across virtual and in-person services [13-15]. Clinicians also remain divided in their satisfaction with telepsychology services [9,10,16,17].

The unified theory of acceptance and use of technology (UTAUT) [18] predicts uptake of new technology into practice from the following 4 factors: (1) performance expectancy, the degree to which a technology is expected to improve performance; (2) effort expectancy, the user’s self-efficacy with the technology; (3) social influence, the perceived norms of technology use; and (4) facilitating conditions, including availability of training and technology fit [18-22]. The UTAUT is based on the theory of planned behavior, wherein attitudes predict intentions, which predict behavior [23]. While the postpandemic behavior of clinicians cannot yet be measured, their intention to use technology serves as a proxy.

The utility of technology may vary considerably depending on whether the clinician is using telephone or videoconferencing. The literature offers comparisons of telepsychology (either telephone or video) [13,24] to in-person services, but few comparisons *between* telepsychology modalities. One meta-analysis indicated that videoconferencing was more effective than telephone for depression and posttraumatic stress disorder in veterans [25], yet a comparison between telepsychology modalities is a major gap in the literature.

This study directly compared the experiences of different modalities (ie, in-person, telephone, and video) and further explored factors pertinent to the uptake of telephone- and video-based services in an AMH setting. Specifically, we predicted the following: (1) Clinicians would have the most positive experiences (ie, satisfaction and therapeutic alliance) with in-person sessions, followed by videoconferencing and then telephone; (2) Clinicians would have a greater intention to use videoconferencing than telephone after the pandemic; (3) Clinicians would have more positive technology attitudes toward video than telephone; and (4) Greater performance expectancy, effort expectancy, social influence, and facilitating conditions would predict greater clinician intentions to use virtual services after the pandemic.

## Methods

### Ethics Approval

This study was approved by the University of Alberta Health Research Ethics Board (Pro00114433).

### Procedures

Secondary data were obtained from a virtual health program evaluation conducted from November 16 to December 21, 2020, using an online survey within the publicly funded AMH service in Alberta, Canada. Clinicians who provided services using both telephone and videoconferencing during the pandemic were included in the sample (n=118; see Table 1 for available sample characteristics). Included clinicians were in AMH practice for 13.7 years on average. They reported seeing 64.1% of their virtual clients previously in-person, with individual virtual sessions being the most frequent format (69.7%), followed by group (47.0%) and couple/family (25.4%) sessions.

**Table 1:** Sociodemographic characteristics of participants.

Characteristic		Value (N=118^a^), n (%)
**Clinician profession**		
	Psychologist	44 (37.3)
	Social worker	22 (18.6)
	Nurse	16 (13.6)
	Occupational therapist	7 (5.9)
	Psychiatrist	6 (5.1)
	Other	22 (18.6)
**Populations served (categories not mutually exclusive)**		
	Children	22 (18.6)
	Adolescents	32 (27.1)
	Young adults	39 (33.1)
	Adults	89 (75.4)
	Older adults	17 (14.4)
	Families	18 (15.3)
**Theoretical orientation**		
	Cognitive behavioral	42 (35.6)
	Integrative/eclectic	23 (19.5)
	Existential/humanistic	6 (5.1)
	Interpersonal/systemic	6 (5.1)
	Other	19 (16.1)
**Prior experience with telepsychology^b^**		
	Do not provide therapy	22 (18.6)
	Telephone	70 (59.3)
	Video	21 (17.8)

^a^Of the total 153 clinicians who completed the survey, 35 used only telephone and were excluded from the analysis to focus on the comparison between telephone and video.

^b^Clinicians who reported “some or quite a lot” were included in this frequency. The remaining participants reported “none or very little.”

### Measures

Modifications to measures are displayed in Multimedia Appendix 1. Table S1 in Multimedia Appendix 1 provides the internal consistencies reported in previous literature and this study. The below measures were repeated across video, telephone, and in-person, where applicable.

The Agnew Relationship Measure-5 (ARM-5) [26] assessed therapeutic alliance in virtual settings on a scale of 1 (strongly disagree) to 7 (strongly agree), and was modified to reflect perceived therapeutic alliance for telephone, video, and in-person sessions in general (ie, across sessions and clients).

Clinicians rated their satisfaction with telephone, video, and in-person sessions on a custom scale of 1 (not at all satisfied) to 10 (very satisfied).

Clinicians’ intentions to use technology after the pandemic were measured by the question, “Given the choice, I would offer telephone [video] sessions,” on a scale of 1 (strongly disagree) to 7 (strongly agree).

The UTAUT-Therapist Version [22] assessed technology acceptance using 13 items based on the original UTAUT, on a scale of 1 (strongly disagree) to 5 (strongly agree), with subscales for performance expectancy, effort expectancy, social influence, and facilitating conditions.

## Results

### Differences Between Telephone and Video Services

Normality assumptions were tested with the Shapiro-Wilk test, and outliers were assessed by boxplots for all analyses. Adjustments based on violated assumptions are described below.

A one-way repeated measures analysis of variance (ANOVA) tested differences in therapeutic alliance among telephone, video, and in-person services. Epsilon (ε=0.850 [27]) adjusted for a violation of the sphericity assumption (χ^2^_2_=22.359; *P*<.001). Therapeutic alliance was significantly different among modalities (*F*_1.700, 197.163_=46.67; *P*<.001; partial η^2^=0.287). Post-hoc analysis with a Bonferroni adjustment revealed that all pairwise differences between modalities were significant (*P*<.001), with in-person services having the greatest therapeutic alliance followed by video services and then telephone services (Table 2).

**Table 2:** Clinicians’ attitudes toward telepsychology variables for in-person, video, and telephone services.

Variable			In-person, mean (SD)		Video, mean (SD)		Telephone, mean (SD)		Difference		95% CI		
											LL^a^		UL^b^
**Satisfaction**			8.97 (1.22)		7.12 (2.33)		5.60 (2.54)						
	In-person/video	N/A^c^		N/A		N/A		1.81^d^		1.19		2.42	
	In-person/telephone	N/A		N/A		N/A		3.36^d^		2.69		4.02	
	Video/telephone	N/A		N/A		N/A		1.55^d^		0.92		2.18	
**Therapeutic alliance**			6.09 (0.98)		5.50 (1.05)		5.18 (1.08)						
	In-person/video	N/A		N/A		N/A		0.57^d^		0.35		0.79	
	In-person/telephone	N/A		N/A		N/A		0.91^d^		0.64		1.19	
	Video/telephone	N/A		N/A		N/A		0.34^d^		0.15		0.54	
**UTAUT^e^ total**			N/A		3.46 (0.61)		3.22 (0.56)		−0.24^d^		−0.36		−0.13
	Effort expectancy	N/A		3.45 (0.95)		3.48 (0.76)		−0.03		−0.13		0.19	
	Performance expectancy	N/A		3.16 (0.78)		2.82 (0.72)		−0.33^d^		−0.49		−0.17	
	Social influence	N/A		3.59 (0.64)		3.18 (0.70)		−0.41^d^		−0.56		−0.27	
	Facilitating conditions	N/A		3.61 (0.68)		3.40 (0.71)		−0.21^d^		−0.34		−0.08	
Intention to use			N/A		4.59 (2.0)		3.62 (2.1)		−0.975^d^		−1.332		−0.617

^a^LL: lower limit.

^b^UL: upper limit.

^c^N/A: not applicable.

^d^*P*<.001.

^e^UTAUT: unified theory of acceptance and use of technology.

A one-way repeated measures ANOVA tested differences between clinicians’ satisfaction with in-person, video, and telephone services. There were significant differences in clinician satisfaction across modalities (*F*_2,228_=82.32; *P*<.001; partial η^2^=0.419; Table 2). Post-hoc analysis with a Bonferroni adjustment revealed that all pairwise differences between modalities were significant (*P*<.001), with in-person services having the greatest satisfaction, followed by video services and then telephone services.

A paired samples *t* test evaluated the difference between clinicians’ intentions to use video and telephone after the pandemic. Clinicians reported significantly greater intention to use video than telephone after the pandemic (t_117_=−5.393; *P*<.001; *d*=0.50; Table 2).

A paired samples *t* test assessed the difference between UTAUT-T total scores for video and telephone. Clinicians reported significantly greater scores for video than telephone (*t*_117_=−4.200; *P*<.001; *d*=0.39). Paired samples *t* tests revealed significant differences in each of the UTAUT predictors in favor of video (*P*≤.001), except effort expectancy (*P*=.75) (Table 2).

### UTAUT Prediction of the Intention to Use

A multiple regression was performed to predict the intention to use video, with concurrent performance expectancy, effort expectancy, social inclusion, and facilitating conditions. The model significantly predicted the intention to use video (*F*_4,113_=14.072; *P*<.001; adjusted *R^2^*=0.31). Performance expectancy was the only unique predictor (*P*<.001) (Table 3).

A multiple regression was performed to predict the intention to use telephone, with concurrent UTAUT factors. The model significantly predicted the intention to use telephone (*F*_4,113_=24.348; *P*<.001; adjusted *R^2^*=0.44). Performance expectancy (*P*<.001), social influence (*P*=.004), and facilitating conditions (*P*=.03) were unique predictors (Table 4).

**Table 3:** Multiple regression results for the intention to use video after the pandemic.

Variable	*B* ^a^	SE *B*^b^	95% CI for *B*			*β* ^e^	*R* ^2^ ^f^		Δ*R*^2^^g^
			LL^c^	UL^d^					
Model^h^	N/A^i^	N/A	N/A	N/A	N/A		0.58	0.31^j^	
Constant	−0.77	1.007	−2.77	1.23	N/A		N/A	N/A	
Performance expectancy	1.16^j^	0.25	0.66	1.7	0.46^j^		N/A	N/A	
Effort expectancy	0.31	0.25	−0.18	0.80	0.15		N/A	N/A	
Social influence	0.28	0.27	−0.26	0.82	0.09		N/A	N/A	
Facilitating conditions	−0.10	0.35	−0.79	0.59	−0.04		N/A	N/A	

^a^*B*: unstandardized regression coefficient.

^b^SE *B*: standard error of the coefficient.

^c^LL: lower limit.

^d^UL: upper limit.

^e^*β*: standardized coefficient.

^f^*R^2^*: coefficient of determination.

^g^Δ*R^2^*: adjusted *R^2^*.

^h^Model: “Enter” method in SPSS Statistics.

^i^N/A: not applicable.

^j^*P*<.001.

**Table 4:** Multiple regression results for the intention to use telephone after the pandemic.

Variable	*B* ^a^	SE *B*^b^	95% CI for *B*			*β* ^e^	*R* ^2^ ^f^		Δ*R*^2^^g^
			LL^c^	UL^d^					
Model^h^	N/A^i^	N/A	N/A	N/A	N/A		0.46	0.44^j^	
Constant	−4.42	0.88	−6.16	−2.67	N/A		N/A	N/A	
Performance expectancy	1.02^j^	0.25	0.51	1.52	0.35^j^		N/A	N/A	
Effort expectancy	0.26	0.24	−0.21	0.74	0.09		N/A	N/A	
Social influence	0.73^k^	0.25	0.24	1.23	0.24^k^		N/A	N/A	
Facilitating conditions	0.57^l^	0.25	0.06	1.07	0.19^l^		N/A	N/A	

^a^*B*: unstandardized regression coefficient.

^b^SE *B*: standard error of the coefficient.

^c^LL: lower limit.

^d^UL: upper limit.

^e^*β*: standardized coefficient.

^f^*R^2^*: coefficient of determination.

^g^Δ*R^2^*: adjusted *R^2^*.

^h^Model: “Enter” method in SPSS Statistics.

^i^N/A: not applicable.

^j^*P*<.001.

^k^*P*=.004.

^l^*P*=.03.

## Discussion

This study explored the differences in clinicians’ perceptions of in-person, video, and telephone services, and the prediction of the intention to use telepsychology after the pandemic. As hypothesized, clinicians had more positive experiences with in-person services, followed by video services and then telephone services across measures. Additionally, clinicians reported greater intention to continue using video services over telephone services. These findings suggest that the perceived utility of technologies varies in AMH care [25], and the merits of and attitudes toward each require consideration when integrating them into routine practice.

Consistent with previous work, this study demonstrated the utility of the UTAUT for predicting the intention to use telephone and video technologies [12,16,22,24]. Specifically, performance expectancy was predictive of the intention to use both video and telephone services, while social influence and facilitating conditions were additionally predictive of the intention to use telephone services. Because telephone services are so different from in-person services (ie, no visual information), the intention to use may be related to how well it fits for a particular service (eg, medication refills [28]) and how much it is supported by the clinician’s profession. Thus, facilitating conditions and social influence may be more relevant to the uptake of telephone services than video services.

The limitations of this study include its cross-sectional design, which prevents causal conclusions. Further, clinicians’ intentions to use telepsychology are a proxy for actual postpandemic technology use. In addition, the study’s small self-selected sample of public AMH clinicians, with underrepresentation of some professions and service settings, limits the generalizability of the findings. For example, our sample did not include urgent settings, where virtual care may present additional challenges [29]. While this study did not include private practitioners, its focus on public health is unique, compared with previous studies focusing primarily on private practice [1,12,16].

In this study, clinicians reported consistently more positive experiences with video services than telephone services, suggesting that the uptake of videoconferencing will face fewer barriers than telephone; however, an overall preference for in-person sessions may result in a return to prepandemic practices. For some clinicians, misgivings about videoconferencing may stem from low performance expectancy. Education on the establishment of a strong therapeutic alliance and the effectiveness of video-based care could decrease clinician hesitancy [3,6,7,30]. Regarding telephone uptake, improved facilitating conditions, such as training, and positive social influence (ie, promotion) may aid uptake. Exploration of how clinicians’ demographic characteristics (eg, age, gender, and prior experience) relate to telepsychology uptake would clarify necessary training or support. For example, those with minimal prior experience may require greater support [24]. In conclusion, while there has been a practice shift to telepsychology during the COVID-19 pandemic, AMH clinicians will likely require ongoing support to maintain this practice change.

## Appendix

Multimedia Appendix 1 Measure customization and reliability coefficients.

## Abbreviations

AMH: addiction and mental health
ANOVA: analysis of variance
UTAUT: unified theory of acceptance and use of technology
