# Supplementary material for: Clinicians’ Attitudes Toward Telepsychology in Addiction and Mental Health Services, and Prediction of Postpandemic Telepsychology Uptake: Cross-sectional Study
Source: JMIR Form Res. 2022 May 13;6(5):e35535. doi: 10.2196/35535 (PMC9143772; doi:10.2196/35535)
Supplement: Multimedia Appendix 1 [file formative_v6i5e35535_app1.docx]

**Multimedia Appendix 1.** Measure customization and reliability coefficients.

**ARM-5**

**The ARM-5 was modified to be used with clinicians rather than clients as in the original scale. In the current study “my therapist was” was replaced with “I felt” or “my clients” and the modality was specified in the instructions.**

Thinking about your **video [telephone; in-person]** sessions in general, please indicate how strongly you agree or disagree with each statement.

**Clinician Satisfaction**

**The following single-item measure was developed by the authors for use in the current study.**

Overall, I am satisfied with providing AMH services by video [telephone; in-person].

| 1 | 2 | 3 | 4 | 5 | 6 | 7 | 8 | 9 | 10 |
| --- | --- | --- | --- | --- | --- | --- | --- | --- | --- |

**Intention to Use Modality in Future**

**The following single-item measure was developed by the authors for use in the current study.**

Thinking about if Covid-19 were not a risk, please indicate how strongly you agree or disagree with each statement about future AMH services you will provide.

| Strongly disagree | Moderately disagree | Slightly disagree | Neutral | Slightly agree | Moderately agree | Strongly agree |  |
| --- | --- | --- | --- | --- | --- | --- | --- |
| 1 | 2 | 3 | 4 | 5 | 6 | 7 |  |

1. Given the choice, I would offer video [telephone] sessions.

**UTAUT-T**

The UTAUT-T was modified to ask about Addiction and Mental Health clinician experiences, rather than only therapists’ experiences while providing therapy. As such, “therapy” was replaced with “session” and “therapist” was replaced with “clinician.” In addition, to compare between video and telephone modalities “online therapy” was replaced with “telephone therapy” and “video therapy” to create a set of questions specifically asking about each modality.

**Table S1.** Cronbach’s Alpha for Current Study Measures.

|  | In-person | Video | Telephone | Previous Studies |
| --- | --- | --- | --- | --- |
|  | α | α | α | α |
|  |  |  |  |  |
| **Therapeutic alliance** | .84 | .89 | .83 | .69 - .82 [23] |
|  |  |  |  |  |
| **UTAUT-T Total** | - | .86 | .81 | .95 [16] |
| Effort Expectancy | - | .84 | .63 |  |
| Performance Expectancy | - | .60 | .55 |  |
| Social Influence | - | .71 | .65 |  |
| Facilitating Conditions | - | .42 | .46 |  |
|  |  |  |  |  |
